# Reply letter to: Is there any importance of Migraine with Aura amongst medical students?

**DOI:** 10.51866/lte.249r

**Published:** 2022-10-17

**Authors:** Anuradha Thiagarajan, Noor Azimah Muhammad, Chai-Eng Tan

**Affiliations:** 1 MMed (Fam Med), Klinik Kesihatan Bukit Minyak, Jalan Bukit Minyak, Bukit Mertajam, Seberang Perai Tengah, Malaysia. Email: anulynx@gmail.com; 2 MMed (Fam Med), Department of Family Medicine, Faculty of Medicine, Universiti Kebangsaan Malaysia, Jalan Yaacob Latif, Cheras, Kuala Lumpur, Malaysia.; 3 MMed (Fam Med), Department of Family Medicine, Faculty of Medicine, Universiti Kebangsaan Malaysia, Jalan Yaacob Latif, Cheras, Kuala Lumpur, Malaysia.

**Keywords:** Migraine Disorders, Students, Medical, Headache

**Dear Editor,**

We have read the letter to the editor entitled ‘Is there any importance of migraine with aura amongst medical students?’^1^ and appreciate the interest in our article. The author has requested for some clarification regarding the epidemiology and clinical profile of migraine amongst medical students.

In our study, the prevalence of migraine and non-migraine headache did not significantly differ according to the year of study. At the time of data collection, the medical school had 296 first-year students, 142 second-year students, 45 fourth-year students and 31 fifth-year students. The majority of the respondents who experienced headache were first- and second-year students. The prevalence of migraine headache was 60.7% (n=51/84) among the first-year students, 64.2% (n=34/53) among the second-year students, 40.0% (n=4/10) among the fourth-year students and 80.0% (n=8/10) among the fifth-year students. Third-year students were not in the campus during data collection. However, there was no significant association found between age and the prevalence of migraine.^2^ Generally, the prevalence of migraine has a bimodal distribution, which peaks in late adolescence to the early 20s and around 50 years of age.^3^ Since our respondents’ age range was narrow (from 18 to 26 years), it was not surprising that age was not significantly associated with the prevalence of migraine.

Regretfully, we did not collect data on the duration of headache and family history of migraine, as it was not within the scope of our research objectives. We used the International Headache Society criteria for migraine as a checklist. The respondents only responded ‘yes’ or ‘no’ to each criterion. Therefore, the duration of headache was not assessed. We also did not specifically ask whether the respondents had a family history of migraine. In future research, these factors could be addressed.

Approximately 66.0% (n=64/97) of our respondents with migraine had aura. Unfortunately, since data were collected in 2013, we were unable to retrieve the descriptive data on the aura symptoms experienced by the respondents. A recent epidemiological study in Korea found that visual aura was present in 24.3% to 29.5% of people with migraine.^4^ Interestingly, the prevalence of visual aura among neurologists with migraine was 51.2%.^5^ This suggests that many individuals with migraine could be unaware of aura symptoms. The higher prevalence among neurologists may be attributed to the fact that they were knowledgeable about visual aura symptoms. Other forms of aura were not well studied.

Taken together, we agree that more local studies on the clinical profile of migraine among Malaysians are needed, although this condition is common.
